# Patterns of Genomic Heterogeneity in a Classic Field Cricket Mosaic Hybrid Zone

**DOI:** 10.1002/ece3.70643

**Published:** 2024-12-01

**Authors:** Luana S. Maroja, Francesca Barradale, Sebastian A. Espinoza‐Ulloa, Steven Bogdanowicz, Jose Andres

**Affiliations:** ^1^ Department of Biology Williams College Williamstown Massachusetts USA; ^2^ Pontificia Universidad Católica del Ecuador Quito Ecuador; ^3^ Department of Biology University of Saskatchewan Saskatoon Saskatchewan Canada; ^4^ Facultad de Medicina Pontificia Universidad Católica del Ecuador Quito Ecuador; ^5^ Department Ecology and Evolutionary Ecology Cornell University Ithaca New York USA

**Keywords:** absolute differentiation, divergence islands, field crickets, gene flow, introgression, speciation

## Abstract

Barriers to gene exchange can be semi‐permeable; some genes are expected to freely flow across species boundaries whereas others, under divergent selection or responsible for reproductive isolation, might not. Genome scans in recently diverged species have identified divergent genomic regions, a pattern that has often been interpreted as islands of restricted introgression in a background of relatively free gene exchange (“genomic islands of speciation”). Areas of high differentiation, most located in the X chromosome (females XX, males X0), have been identified in the hybridizing field crickets *Gryllus firmus* and *Gryllus pennsylvanicus* . These species were assumed to follow an islands of speciation model, with highly differentiated areas interpreted as areas of reduced introgression. We sequenced the *G. firmus* genome to localize previously studied SNPs and sample a larger area around them in 8 allopatric populations (4 of each species). We use these data to test expectations for the islands model, in which non‐introgressing areas should have both high absolute and relative differentiation. We find that in the allopatric populations, the areas with high relative differentiation (mostly X‐linked), previously interpreted as non‐introgressing, do not have high absolute differentiation as would be expected under the “islands model.” We also show that the estimated divergence time based on nuclear DNA is about 4× older than that estimated based on mtDNA (800 K vs. 200 K years ago). We discuss the implications of our results for introgression into allopatric populations.

## Graphical abstract

We examine patterns of introgression between *Gryllus firmus* and *Gryllus pennsylvanicus* allopatric populations. We show that previously classified “non‐introgressing” loci map mainly to the X chromosome and do not show higher differentiation between species as would be expected under an “islands of speciation” model. We discuss the implications of this result to the understanding of this classic mosaic hybrid zone.

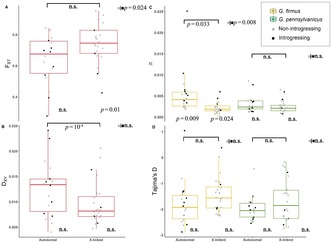

## 1 Introduction

Characterizing the genomic architecture of barriers to gene exchange is an essential step in understanding speciation. While rapid advances in sequencing technology have allowed us to investigate entire genomes across the speciation continuum, our understanding of the link between genome‐wide patterns of divergence, phenotypes, and reproductive isolation is still quite limited. If barriers to gene exchange are a result of evolutionary forces acting on individual genes (genic view of speciation; Wu 2001) then, in species still exchanging genes, alleles that are neutral or globally advantageous will be able to cross species boundaries, whereas genome regions under divergent selection or responsible for reproductive isolation will not. The result is that species boundaries can be semi‐permeable (Barton and Hewitt 1981; Harrison 1990; Wu 2001), and in some instances, only a few loci may be essential for the maintenance of species isolation (e.g., Barr and Fishman 2010; Dasmahapatra et al. 2012; Dopman et al. 2005; Machado and Hey 2003; Nosil and Schluter 2011). These loci might act as “divergence centers” (Charlesworth, Nordborg, and Charlesworth 1997), building up additional divergence because of reduced recombination rates around them (divergence hitchhiking) and eventually leading to a clustering of loci contributing to differentiation (Charlesworth, Nordborg, and Charlesworth 1997; Feder and Nosil 2010; Strasburg et al. 2012; Yeaman and Whitlock 2011). Indeed, genome scans have identified divergent genomic regions in many recently diverged, hybridizing species (e.g., Carneiro et al. 2014, 2010; Hirase et al. 2021; Nadeau et al. 2012; Nosil, Funk, and Ortiz‐Barrientos 2009; Via and West 2008), a pattern that has often been interpreted as islands of restricted introgression in a background of relatively free gene exchange (“genomic islands of speciation,” Malinsky et al. 2015; Sun et al. 2022; Turner, Hahn, and Nuzhdin 2005 and “genomic islands of differentiation or divergence,” Harr 2006; Nosil, Funk, and Ortiz‐Barrientos 2009; Quilodran et al. 2020).

In many studies, *F*_ST_, the fixation index, has been used as the measure of divergence and, in these cases, “islands of differentiation” represent genome regions with high *F*_ST_ (“outliers”). Explicit models of divergence with gene flow have explored how and whether divergence hitchhiking and/or “genomic hitchhiking” can produce such “*F*_ST_ outliers.” The assumption is that in early divergence, gene flow homogenizes most genome regions, but regions that contain genes that contribute to local adaptation or reproductive isolation will remain differentiated (resistant to introgression). However, this “divergence with gene flow” interpretation has been challenged (Cruickshank and Hahn 2014; Nachman and Payseur 2012; Quilodran et al. 2020; White et al. 2010), because a pattern of high relative differentiation (e.g., based on allele frequency differences or *F*_ST_) on a background of low differentiation can be produced even in the complete absence of gene flow. High *F*_ST_, island‐like patterns can also result from loss of variation within species (e.g., caused by a recent selective sweep), as well as by increased divergence between species (e.g., caused by lack of gene flow and independent evolutionary trajectories). Hence, genome regions that have similar allele frequencies may reflect persistence of shared ancestral polymorphism, with divergent regions being the result of local selective sweeps.

The two alternative scenarios, differential introgression and no introgression/local selection, can be distinguished with measurements of absolute genetic divergence. In the impermeable model (no introgression), species‐specific selective sweeps will convert intra‐specific variation into fixed differences, increasing relative genetic differentiation. These high *F*_ST_ regions, however, are not expected to have high levels of absolute genetic differentiation (e.g., *D*_XY_) in relation to the rest of the genome, given that all areas were already at a base level of absolute genetic divergence (dependent on time since divergence and mutation rate). Conversely, in a semi‐permeable model (differential introgression), certain loci containing introgressed segments are selected against (impermeable regions), but other background loci with introgressed segments are not (permeable regions). We then expect high levels of *F*_ST_ to be associated with elevated *D*_XY_ values, since the genome‐wide *D*_XY_ will decrease due to mixing of permeable loci. Cruickshank and Hahn (2014) have demonstrated that for many of the classic examples of heterogeneous genomic divergence, genome regions with high *F*_ST_ do not have correspondingly high values of *D*_XY_ (as would be expected in the semi‐permeable model) and thus might not represent “islands of differentiation.” Here we study a classic cricket mosaic hybrid zone, examining the question of introgression into allopatric population and whether genomic divergence patterns follow the “islands” model.

The two sister species of field crickets, *Gryllus firmus* and *G. pennsylvanicus* , form an extensive and well‐characterized hybrid zone in eastern North America for which previous data based on relative genetic differentiation and patterns across the hybrid zone pointed to differential introgression (Larson, Becker, et al. 2013; Larson et al. 2012), where X‐linked loci were the most “resistant to introgression” (Gainey, Kim, and Maroja 2018; Maroja et al. 2015).

**FIGURE 1:**
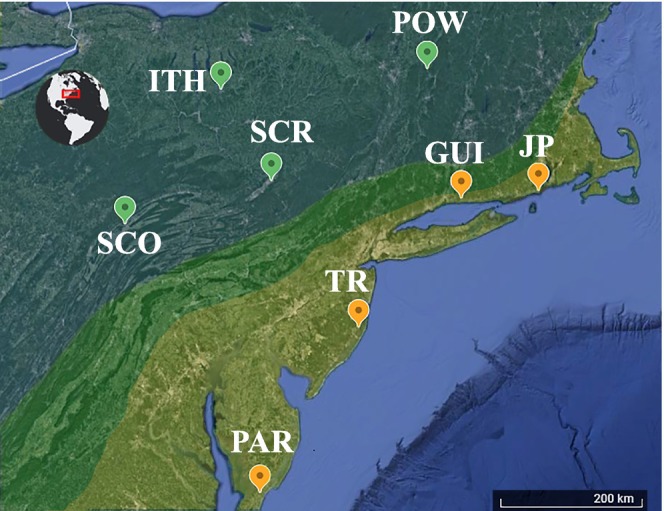
Collection localities for the 96 sequenced individuals across four allopatric populations of each species (*n* = 12 individuals per population). The highlighted areas correspond to the approximate distribution of each species: Yellowish for *Gryllus firmus* , blue‐green for *G. pennsylvanicus* and the approximate location of the hybrid zone is shown in green.

While the estimated mtDNA divergence time is only about ~200 K years (Broughton and Harrison 2003; Maroja et al. 2009), they exhibit multiple barriers to gene exchange including habitat isolation (Larson, Becker, et al. 2013; Rand and Harrison 1989; Ross and Harrison 2002, 2006), temporal isolation (Harrison 1985), behavioral and cuticular hydrocarbon differences (Heggeseth et al. 2020; Maroja et al. 2009, 2014) and a post‐mating, prezygotic barrier in which male *G. pennsylvanicus* fail to fertilize *G. firmus* eggs (Harrison 1983; Larson et al. 2012). The last barrier results in a unidirectional reproductive incompatibility (only *G. pennsylvanicus* females produce F1 hybrids). A priori, the genomic pattern of differentiation between the two species follows a pattern consistent with a genomic islands model: Most previously analyzed SNPs showing major allele frequency differences between allopatric populations and restricted introgression are concentrated on only two regions, the X chromosome (females are XX and males X0) and in a small window on one autosome (LG14) (Gainey, Kim, and Maroja 2018; Maroja et al. 2015). An observation also consistent with the X chromosome playing a prominent role in reproductive isolation (e.g., Charlesworth, Coyne, and Barton 1987; Good, Dean, and Nachman 2008; Hu and Filatov 2016; Masly and Presgraves 2007; Meisel and Connallon 2013). However, in these previous studies, the absolute divergence was not calculated for any of the SNP locations, and cline calculations were conducted prior to mapping, thus based on a diploid model (i.e., not taking into account X linkage). In this paper we calculate *D*_XY_ data for ~5 kbp regions around 48 of the previously characterized SNPs (Gainey, Kim, and Maroja 2018; Larson, Becker, et al. 2013; Larson et al. 2014; Maroja et al. 2015) across four allopatric populations of each species (Figure 1). We also assemble a draft genome, report genes located around these previously characterized SNPs, and recalculate divergence times using nuclear DNA data and outgroup species.

We fail to observe an elevated *D*_XY_ in areas of elevated *F*_ST_ or any higher *D*_XY_ for previously characterized non‐introgressing loci and conclude that patterns of divergence in allopatric populations are more consistent with a lack of introgression. Furthermore, according to our genomic‐based divergence calculations, these two cricket species diverged ~800 K years ago, substantially earlier than indicated by previous mtDNA‐based estimates (~200 K years ago). We discuss the significance of these findings for the understanding of this hybrid zone.

## 2 Methods

### 2.1 Reference Genome Sequencing, Assembly, Gene Model Prediction and Annotation

We extracted high molecular weight genomic DNA from a single wild‐caught *G. firmus* male cricket collected in Guilford, CT (41°16′07″; −72°40′02″) using a MagAttract HMW DNA Kit (Qiagen). We then prepared a 10× Genomics library using the Chromium Gel Bead and Library Kit (10× Genomics, Pleasanton, CA, USA) and the Chromium instrument (10× Genomics) following the manufacturer's protocols. The 10× library was distributed in four lanes for barcoding and sequenced at the Cornell Sequencing center (BRC) on a NextSeq500 (150 bp PE). Then, we assembled the sequences using the Supernova assembler ver. 1.2.2 under default parameters and the supernova parameter—style = pseudohap to obtain a single consensus sequence for each pair‐ended scaffold (Weisenfeld et al. 2017).

We used the MAKER genome annotation pipeline (ver. 3, Cantarel et al. 2008) to annotate the scaffolds. First, we characterized repetitive elements using two approaches, de novo identification and reference‐based identification. For de novo identification, we used RepeatModeler Open‐1.0 (Smit and Hubley 2008–2015), which created a database of 1576 repetitive families. We used RepeatMasker Open‐4.0 (Smit, Hubley, and Green 2013–2015) for reference‐based repeat identification using the reference database for Arthropoda and Insecta clades (one run for each clade). We implemented both of these processes on the NCBI search engine. We then created a single GFF file (ProcessRepeats, RepeatMasker command) with all identified repeats. Second, to guide our gene model prediction, we created a custom reference protein database with 562,554 proteins (560,292 from SwissProt <including isoforms> and 2252 *Gryllus* spp. proteins recovered from NCBI). Additionally, we used the published transcriptome from *Gryllus rubens* as reference (Berdan et al. 2016). Finally, with these information sources (model repetitive regions and gene evidence), we carried out the annotation and gene prediction with MAKER Annotation (Cantarel et al. 2008) as is described in the flow‐chart in appendix (DRYAD Figure 1).

The draft genome data were used to locate previously characterized SNPs (Larson, Becker, et al. 2013; Larson et al. 2012) and design primers spanning ~5 kbps around each SNP. We did not obtain population level data from the *G. firmus* draft genome.

### 2.2 Field Sampling

We sequenced 96 individuals from four *G. firmus* and four *G. pennsylvanicus* populations (12 individuals each, Figure 1). The *G. firmus* populations were from Tom's River, NJ (TR, 39°45′00″; −74°11′33″); Judith Point, RI (JP, 41°21′38″; −71°28′53″); Guilford, CT (GUI, 41°16′07″; −72°40′02″); and Parksley, VA (PAR, 37°45′58″; −75°36′00″). The *G. pennsylvanicus* populations were from Ithaca, NY (ITH, 42°26′01″; −76°29′59″); State College, PA (SCO, 40°47′59″; −77°52′05″); Scranton, PA (SCR, 41°24′25″; −75°35′46″); and Pownal, VT (POW, 42°45′16″; −73°14′02″), see map on Figure 1. Most of the *G. firmus* and *G. pennsylvanicus* populations were collected by L. S. Maroja in 2004–2005, the Pownal, VT and the RI populations were collected in 2010. In addition, we also sequenced four outgroup individuals: two *G. rubens* (from Durhan, NC) and two *G. bimaculatus* (from a captive colony, Hoy lab at Cornell).

### 2.3 Targeted Resequencing and Individual Genotype Reconstruction

**FIGURE 2:**
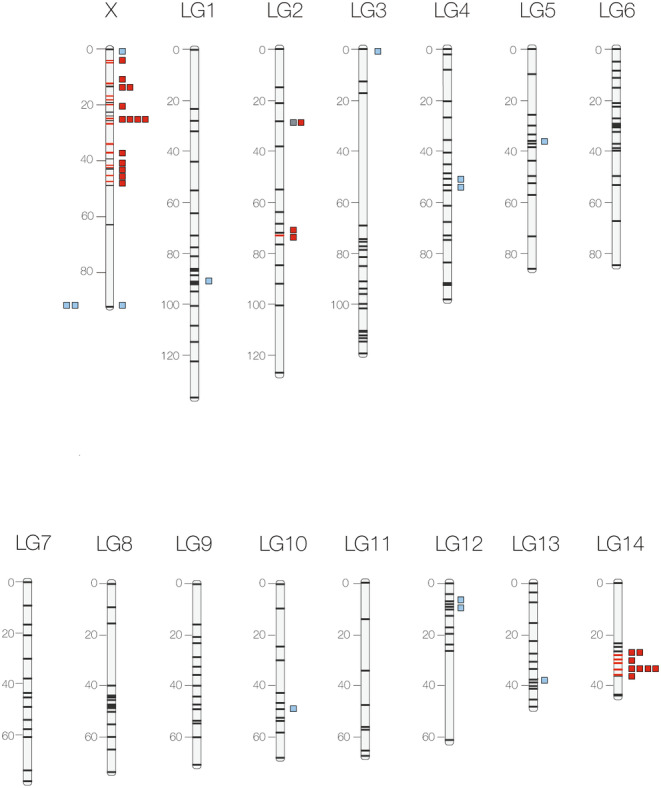
Cricket genetic map based on Gainey, Kim, and Maroja (2018) for the X chromosome and Maroja et al. (2015) for autosomes. Sequenced loci are shown as blue squares for “introgressing” and red squares “non‐introgressing” according to Larson, Andres, et al. (2013) and Larson et al. (2014). The locus in gray (LG2, locus 202) was not previously characterized as introgressing or non‐introgressing. The two blue squares outside the X chromosome, were originally located to the X in Maroja et al. (2015) but were not able to be finely mapped in Gainey, Kim, and Maroja (2018) and therefore might not be located on the X chromosome.

To design PCR primers, we located the scaffolds containing matches to previously sequenced loci (Gainey, Kim, and Maroja 2018; Larson, Andres, et al. 2013; Larson et al. 2014; Maroja et al. 2015). We sampled these previously characterized introgressing and non‐introgressing loci from autosomes and X chromosomes; most non‐introgressing loci were located on the X chromosome, the few autosomal non‐introgressing loci were located on LG14 (Figure 2 shows the genetic map position of our sampled loci). We used BatchPrimer3 (You et al. 2008) to design primers spanning ~5–8 kb around previously introgressing (*n* = 13) and non‐introgressing loci (*n* = 34) and one locus not previously studied located on either the X (*n* = 27) or the autosomal chromosomes (*n* = 21). We also sequenced a single locus that was not previously characterized (locus 202). To amplify the loci, we used LongAmp Hot Start *Taq* DNA Polymerase (New England Biolabs) following the recommended protocol and annealing temperature of 55°C. We pooled the 49 PCR products for each individual and digested 5ul of individual samples with dsDNA fragmentase (New England Biolabs). We then purified digested samples with 1× Agencourt AMPureXLbeads (BeckmanCoulter Inc.) and re‐suspended each sample in 15 μL of 0.5× AE buffer. Before adding barcodes to fragmented individual products, we blunted the DNA ends using T4 Polynucleotide Kinase (New England Biolabs) and then adenylated fragments using Taq polymerase. We then ligated Illumina Truseq adapters with T4 DNA ligase to each individual sample. To identify each individual, we added dual TruSeq barcodes with PCR (OneTaq Hot Start, NEB). We then pooled individuals and cleaned up the reaction with 0.7× Agencourt AMPureXP beads (BeckmanCoulter Inc.) and resuspended the pooled fragments into 0.3× AE buffer and 0.1× Tween detergent (concentration of 2–5 nM DNA). The library was sequenced with a NextSeq 500, 2 × 75 paired reads.

After removing low quality bases in Geneious R11.1.5 (Biomatters), we assembled the reads to the 49 known genomic reference sequences, one individual at a time (“Map to reference” workflow with medium‐low sensitivity). We produced one consensus file for each locus by individual combination. First the reads for each individual/locus were aligned to a reference, then consensus sequences were called if there was a minimum coverage of > 8 reads; nucleotide positions were called heterozygous if the minimum allele had at least 25% frequency. Finally, we aligned all individual consensus files for each locus, creating a FASTA alignment file which included all individual sequences for each of the loci.

To export the sequences, we first removed all gaps by using the Mask Alignment tool, then we converted poor quality triple ambiguous sites D, H, V, B, into N (i.e., missing data—notice that ambiguous sites representing heterozygosity were kept) and imported individual locus files into DNAsp v.6 (Rozas et al. 2017) and unphased haplotypes with the default parameters in PHASE and individuals separated by species ( *G. firmus* and *G. pennsylvanicus* ). Because this method separates the male loci into two identical reads, we removed one X‐linked read from each male individual. To identify males (individuals were too young to be morphologically sexed), we screened five high coverage X‐linked loci for homozygosity, and classified homozygous individuals as male. To account for sequencing error, if an individual had only a few (3 or fewer) polymorphisms, we checked these polymorphisms for allele quality and frequency before assigning a sex to the individual.

### 2.4 Divergence Analyses

We used DnaSP v.6 (Rozas et al. 2017) to calculate Tajima's *D* and *π* as well as *D*_XY_ and *F*_ST_ (Lynch and Crease 1990) between species using the “Divergence between populations” and “Gene Flow and Genetic Differentiation” commands, respectively. To test if the obtained values were significantly different between contrasting classes of loci (e.g., non‐introgressing vs. introgressing; autosomal vs. sex‐linked), we used a permutation analysis (permutation of values for whole individual/locus sequences) as implemented in the Boot package of R using 10,000 random reallocations.

### 2.5 Estimates of Divergence Time

We used the outgroups *G. bimaculatus* and *G. rubens* to calculate a rough mutation rate for each of the 48 loci as an initial input for IMa3 (Hey et al. 2018). To do this we first calculated the mtDNA (COI) divergence times between *G. firmus* / *G. pennsylvanicus* and the outgroups using sequences available in Genbank. We used sequences KC488896–KC489085 for *G. firmus* and *G. pennsylvanicus* (Larson, Becker, et al. 2013), sequences KR071876, KU705555, KY646234, MF046161 for *G. bimaculatus* and sequences AY234789–AY234792 for *G. rubens* . We used D=2μt, where *D* is the average divergence between consensus sequences of two species calculated with MEGA X (Kumar et al. 2018), *μ* is the mutation rate in percent difference per million years average arthropod mitochondrial DNA mutation rate set to the standard 0.0115 per site/My according to Brower (1994), also see Pons et al. (2010) and Chang et al. (2020), and *t* is the divergence time. Using our estimated mtDNA divergence time between the ingroup and each of outgroups (3.2 million years for *G. bimaculatus* and 2.7 million years for *G. rubens* ), we then estimated mutation rates for each locus given the average *D* between ingroup and outgroup and the divergence time estimated from mtDNA (i.e., 3.2 million years for *G. bimaculatus* and 2.7 million years for *G. rubens* ). When both outgroups ( *G. rubens* and *G. bimaculatus* ) were available (31/48 loci) we used the average mutation value as input for IMa3 (Hey et al. 2018). A total of 8/48 loci did not have any outgroup sequences available; we did not input a mutation rate for these loci (list of mutation rates and IMa3 input files can be found on DRYAD). As we point out in the discussion, a potential caveat of our approach to estimate nuclear divergence time is that it still relies on the standard mtDNA evolution rate (Brower 1994) for the estimation of initial mutation rate value for IMa3. To satisfy the requirement of no‐recombination, we used only the longest non‐recombining region for each locus, identified with the IMgc software package (Woerner, Cox, and Hammer 2007). We assumed one generation per year, as *Gryllus* in the Northeast of the US survive the winter exclusively through the diapause egg stage and have non overlapping generations. We assumed infinite sites model (I), informed the autosomal or X chromosomal location of each locus (DRYAD Table 1 contains detailed information on size and mutation rate for each locus) and assumed a population size prior of 40 (4 Nμ), time since speciation of 90 (tμ), and migration rate of 4 (m/μ). The parameters reached stationarity (no perceivable trends) after about 350,000 generations with a geometric heating scheme of 100 parallel hot chains (hn = 100, ha = 0.99, hb = 0.75). After 400,000 generations of burn‐in, we collected an additional 30,000 genealogies. We ran simulations with these parameters three times and in all cases, we obtained equivalent results.

**TABLE 1:** Summary of *F*_ST_ and *D*_XY_ results (also see Figure 3). Comparisons across non‐introgressing and introgressing loci located on autosomes or X chromosome (*n* loci represents the number of analyzed loci in each category).

	*F*_ST (median)_	*D*_XY (median)_	*n* loci	*p*_Random_ (*F*_ST_/*D*_XY_)
Non‐introgressing/introgressing	0.75/0.64	0.008/0.01	30/12	**0.024**/0.060
Autosomal/X‐linked	0.67/0.74	0.013/0.008	18/24	0.135/**10** ^**−4**^
Autosomal only (non‐introgressing/introgressing)	0.67/0.76	0.012/0.014	10/8	0.37/0.87
X‐linked only (non‐introgressing/introgressing)	0.75/0.64	0.0080/0.0082	4/20	**0.024**/1

*Note: p*_Random_ represents the value of permutation analysis using 10,000 random reallocations for *F*_ST_ comparisons and for *D*_XY_ comparisons, significant results are in bold.

### 2.6 Phylogeny Reconstruction

To build the phylogeny, we first created a concatenated sequence file of all loci, using the command “concat” in the program seqkit (Shen et al. 2016) and realigning data in Geneious R11.1.5 (Biomatters). We eliminated 10 loci that had fewer than 65 individuals sequenced (loci 432, 202, 726, 1121, 4205, 7164, 14741, 8026, 5556, and 6271) and eliminated three loci which did not have any outgroups (loci 211, 3968, and 5961). We also eliminated 11 *G. firmus* , 8 *G. pennsylvanicus* , one *G. bimaculatus* and one *G. rubens* individuals which had fewer than 80% data completion. The final dataset was 172,357 nucleotides long (34 loci) and contained 37 *G. firmus* , 40 *G. pennsylvanicus* , one *G. rubens* , and one *G. bimaculatus* . We used MrBayes version 3.2.6 (Huelsenbeck and Ronquist 2001) with the general time reversible model with invariant sites, gamma rates, and default priors (GTR + I + G), allowing the rate at each site to change over evolutionary history. We ran 5 million generations and discarded the first 25% of the trees. We also ran neighbor‐joining and maximum likelihood methods, but since these trees were virtually identical to the Bayesian tree, we omitted these results.

## 3 Results

The gDNA used for 10× Genomics DNA library construction had a high molecular weight (HMW) of 38.6 kb which generated 772.7 million raw pair‐end reads (61× coverage). After trimming, these reads had an average length of 139.5 bp; ~76% of these reads had a *Q* > 30. The percentage of non‐duplicated, phased reads was ~45%. These data are within the optimal standard values suggested by Supernova Assembler software manufacturer.

Supernova assembled 14,450 long scaffolds (over 10 kb) having a total length of 1.28 Gb—much smaller than the 1.66 Gb *Gryllus bimaculatus* genome (Ylla et al. 2021), likely due to incompletion. The average distance between SNPs (hetdist) was ~122 bp. The contig N50 and the scaffold N50 of the assembled genome were 22.73 kb and 346.11 kb, respectively. All statistic outputs from Supernova analyses can be found in the DRYAD, Table S2.

For the 48 individually sequenced loci (Figure 1, population locations), we accepted a minimum depth per individual of 8 reads and, because of low coverage, some individual/loci combinations were excluded; the resulting average number of individuals per locus was 77 ± 22 individuals. Of these loci, 13 are introgressing and 34 are non‐introgressing, and one was not determined based on previous cline analysis (Larson, Andres, et al. 2013). Of the 48 loci, 21 were autosomal and 27 were X‐linked (Gainey, Kim, and Maroja 2018; Maroja et al. 2015).

### 3.1 Gene Annotation

MAKER *de novo* Annotation established the homologous location of 5064 genes (mean length ~4.23 kb). Almost 98% of the annotations were within 0.5 AED score (Annotation Edit Distance), suggesting a high accuracy in the prediction of gene models based on homology. To adjust the homology‐based gene models, and to predict unidentified genes we used SNAP (Korf 2004) and AUGUSTUS (Stanke and Waack 2003). SNAP was trained through two consecutive runs, after which we obtained 19,501 genes (mean length ~5.78 kb). Over 84% of the SNAP‐based gene models had AED scores < 0.5. AUGUSTUS was trained and ran independently, resulting in 19,243 genes (mean length ~6.72 kb). Around 84% of the AUGUSTUS‐based gene models showed AED score < 0.5. The final annotation, carried out combining all results from the homology, SNAP, and AUGUSTUS based gene models, resulted in 19,157 gene models (mean length ~7.12 kb), 84% of them with AED score < 0.5.

The median coding sequence (CDS) length of the final annotation was 939 base pairs (bp), and the median transcript length was 522 bp. Furthermore, we observed that the 5′ untranslated regions (UTR) had a median length of 34 bp, and the 3′ UTR had a median length of 177 bp. The analyses revealed a total of 19,158 genes and 19,157 transcripts, with 18,885 unique transcripts and 12,499 unique CDS among them, showcasing a diverse transcriptional landscape. Of these genes, 301 genes exhibited a 5′ UTR, and 37 genes had a 3′ UTR.

We identified all annotated genes located in scaffolds containing each of the 48 ~5 kb loci of interest. Within these regions we found a total of 501 annotated genes (DRYAD contains the gff files). Thirty‐seven of these regions contained annotated coding genes inside the target locus sequence, with five of them containing more than one gene.

### 3.2 Relative ( *F*_ST_ ) and Absolute (*D*_XY_) Divergence Patterns

For the divergence analyses, we excluded six loci (202, 1121, 5556, 6271, 7164, and 8026) that had a low number of represented populations (in some cases all individuals of a given population were excluded due to low coverage). We classified loci as “introgressing” or “non‐introgressing” according to Larson, Andres, et al. (2013) and Larson et al. (2014). We used a total of 42 loci, 12 of them introgressing (8 autosomal and 4 X‐linked) and 30 non‐introgressing (10 autosomal and 20 X‐linked). The estimated sex ratio for *G. firmus* was 25:23 (♂:♀) and for *G. pennsylvanicus* it was 30:18 (♂:♀).

**FIGURE 3:**
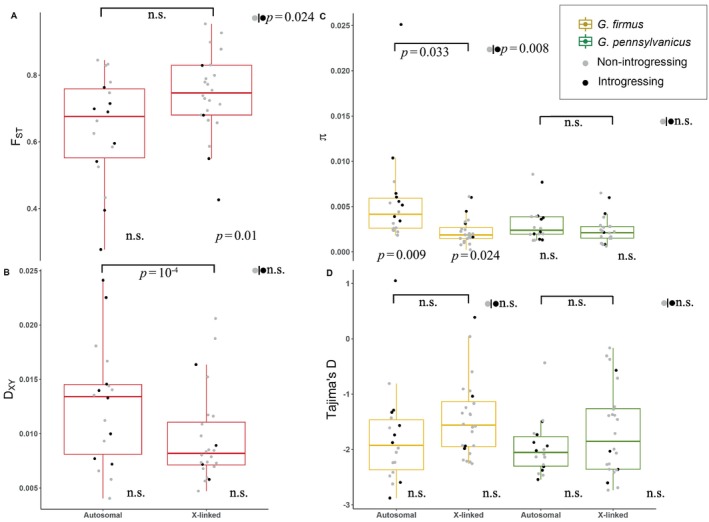
Box plots and data points for *F*_ST_ (A), *D*_XY_ (B), *π* (C) and Tajima's *D* (D). Between species *F*_ST_ and *D*_XY_ comparisons (A, B) are shown in red; within species *π* and Tajima's *D* are shown in yellow for the beach cricket, *Gryllus firmus* , and green for the field cricket, *G. pennsylvanicus* . Data points for previously characterized introgressing loci are shown in black, and for non‐introgressing loci are shown in gray. Significant values for comparisons between autosomes and X chromosomes are shown on top; overall comparisons (without taking into account chromosomal linkage) between introgressing and non‐introgressing loci are shown to the right of each group, sidewise to gray and black circles; comparisons between introgression and non‐introgressing loci taking into account chromosomal linkage are shown on the bottom right of each box plot. For sample sizes refer to the text (Section 3).

As expected, loci showing reduced introgression in previous genomic cline analyses (Larson, Andres, et al. 2013; Larson et al. 2014) showed elevated values of relative divergence (median *F*_ST non‐introgressing_ = 0.75, *F*_ST introgressing_ = 0.64, *p*_Random_ = 0.024). However, most of the loci (20/30) in the non‐introgressing category are located in the X chromosome, so the observed pattern could be driven by chromosomal linkage rather than previously characterized “introgression patterns”—that is, loci could be classified as “non‐introgressing” due to X‐linkage and not because they reduce hybrid fitness. Two methodological problems—males being classified as homozygotes (for the X‐linked loci), and the one‐way directional incompatibility observed in this hybrid zone—could increase the probability of X loci being classified as non‐introgressing. Indeed, taking into account chromosomal linkage (median *F*_ST autosomal_ = 0.67, *F*_ST X‐linked_ = 0.74, *p*_Random_ = 0.135, Figure 3A), we found no clear association between *F*_ST_ and the previously measured introgression pattern per se: for the autosomal loci, there was no relationship between *F*_ST_ and introgression pattern (median *F*_ST autosomal non introgressing_ = 0.70, *F*_ST autosomal introgressing_ = 0.76, *p*_Random_ = 0.37, Figure 3A). In the X chromosome, introgressing loci do indeed seem to have lower *F*_ST_ values (median *F*_ST X‐non introgressing_ = 0.75, *F*_ST X‐introgressing_ = 0.64, *p*_Random_ = 0.024). We note that this result is based on few introgressing loci (*n* = 4, Figure 3A), including two outliers that might not be X‐linked (unconfirmed linkage). For summary of results see Table 1.

In contrast with the predictions of the genomic island model, non‐introgressing loci did not show elevated *D*_XY_ values (median *D*_XY non‐introgressing_ = 0.008, *D*_XY introgressing_ = 0.01, *p*_Random_ = 0.060, Figure 3B). While the median *D*_XY_ between these two categories is not significant, the difference goes in the *opposite* direction from the expectations of the genomic islands model (according to the islands model, loci showing reduced introgression should have higher, not lower, *D*_XY_ value). There was a large difference between median autosomal and X‐linked *D*_XY_ (*D*_XY autosomal_ = 0.013, *D*_XY X‐linked_ = 0.008 *p*_Random_ = 10^−4^, Figure 3A). Taking into account chromosomal linkage, we found no relationship between *D*_XY_ and introgression pattern (median *D*_XY autosomal non introgressing_ = 0.012, *D*_XY autosomal introgressing_ = 0.014; *p*_Random_ = 0.87, Figure 3B and median *D*_XY X‐non introgressing_ = 0.0080, *D*_XY X‐introgressing_ = 0.0082, *p*_Random_ = 1). Furthermore, we found no significant correlation between *F*_ST_ and *D*_XY_ (*ρ*_Spearman_ = 0.167, *p*_bootstrap_ = 0.13).

### 3.3 Neutrality Tests and Nucleotide Diversity

The genomic baseline value of Tajima's *D* can be biased by demographic effects particularly after population expansions or contractions. As expected, due to likely demographic expansion after the last glacial maxima (in late Pleistocene epoch), both species had a negative median Tajima's *D* ( *G. firmus* : −1.65 CI_BCa‐95%_ (−1.96, −1.43); *G. pennsylvanicus* : −1.9, CI_BCa‐95%_ (−2.19, −1.81), Figure 3D), with the signal especially strong in *G. pennsylvanicus* , the species with broadest current geographic distribution. For both species, the median Tajima's *D* was almost identical between introgressing and non‐introgressing loci ( *G. firmus* : *T*_D introgressing_ = −1.65, *T*_D non introgressing_ = −1.64, *p*_Random_ = 0.97; *G. pennsylvanicus* : *T*_D introgressing_ = −2.02, *T*_D non introgressing_ = −2.00, *p*_Random_ = 0.90).

In terms of genetic variation, the median *π* for *G. firmus* was substantially smaller for non‐introgressing than for introgressing loci, while *G. pennsylvanicus* had almost identical median *π* values for introgressing and non‐introgressing loci ( *G. firmus* : *π*_introgressing_ = 0.054, *π*_non introgressing_ = 0.002, *p*_Random_ = 0.008; *G. pennsylvanicus* : *π*_introgressing_ = 0.0029, *π*_non introgressing_ = 0.0020, *p*_Random_ = 0.158). Taking into account chromosomal linkage (autosomal or X‐linked) we found no significant differences between introgressing and non‐introgressing loci in *π* or Tajima's *D* for *G. pennsylvanicus* . For *G. firmus* there were differences between introgressing and X‐linked loci located in autosome or X‐linked (*π*_autosomal introgressing_ = 0.0058 *π*_autosomal non‐introgressing_ = 0.0026, *p*_Random_ = 0.009) (*π*_X introgressing_ = 0.0038, *π*_X non‐introgressing_ = 0.0018, *p*_Random_ = 0.024).

While in *G. pennsylvanicus* X‐linked and autosomal loci have similar median levels of variation (*π*) and Tajima's *D* values (*π*_autosomal_ = 0.0024, *π*_X‐linked_ = 0.0021, *p*_Random_ = 0.64; *T*_D autosomal_ = −2.052, *T*_D X‐linked_ = −1.853, *p*_Random_ = 0.64; Figure 3C,D), in *G. firmus* X‐linked loci showed median levels of variation slightly reduced, but not significantly so, ( *G. firmus* : *π*_autosomal_ = 0.0041, *π*_X‐linked_ = 0.0018, *p*_Random_ = 0.033, Figure 3C). This reduced variation on the *G. firmus* X‐chromosome was not associated with more negative Tajima's *D* values ( *G. firmus* : *T*_D autosomal_ = −1.92, *T*_D X‐linked_ = −1.56, *p*_Random_ = 0.144, Figure 3D).

The autosome with a large number of previously identified non‐introgressing loci (LG14, all genes contained in a 7 cM window, Figure 2) did not show lower genetic diversity in either in *G. pennsylvanicus* (*π*_LG14_ = 1.9 × 10^−3^, *π*_all other autosomal_ = 2.6 × 10^−3^, *p*_Random_ = 0.679) or in *G. firmus* (*π*_LG14_ = 2.6 × 10^−3^, *π*_all other autosomal_ = 5.1 × 10^−3^, *p*_Random_ = 0.139).

### 3.4 Estimates of Divergence Time

To calculate divergence time, we used 48 loci. The loci had average and standard deviation non‐recombining length of 3584.4 ± 1013.9 bp (original untrimmed length 4760.77 ± 103.319) and average and standard deviation mutation rate of 4.052 × 10^−9^ ± 1.301 × 10^−9^ (DRYAD Table S1). As expected, due to lack of recombination in males, the trimmed (non‐recombining) sequence length was larger for X‐linked than autosomal loci (4001.89 and 3047.67 respectively, *t* = 3.5, *p* < 0.001); the difference was only marginally significant for the original total sequence length (4800.1 and 4730.7, respectively, *t* = 2.1, *p* < 0.044). There was no difference in mutation rate between X‐linked and autosomal loci (4.00 × 10^−9^ and 4.10 × 10^−9^, *t* = 0.2, *p* > 0.8). For the loci previously classified as “introgressing” or “non‐introgressing” there were no differences in either total length (4743.231 and 4779.629 respectively, *t* = −1.22, *p* > 0.2), non‐recombining trimmed length (3293.301 and 3692.54 respectively, *t* = −1.28, *p* > 0.2) or mutation rate (4.15 × 10^−9^ and 4.00 × 10^−9^ respectively, *t* = 0.33, *p* > 0.74).

**FIGURE 4:**
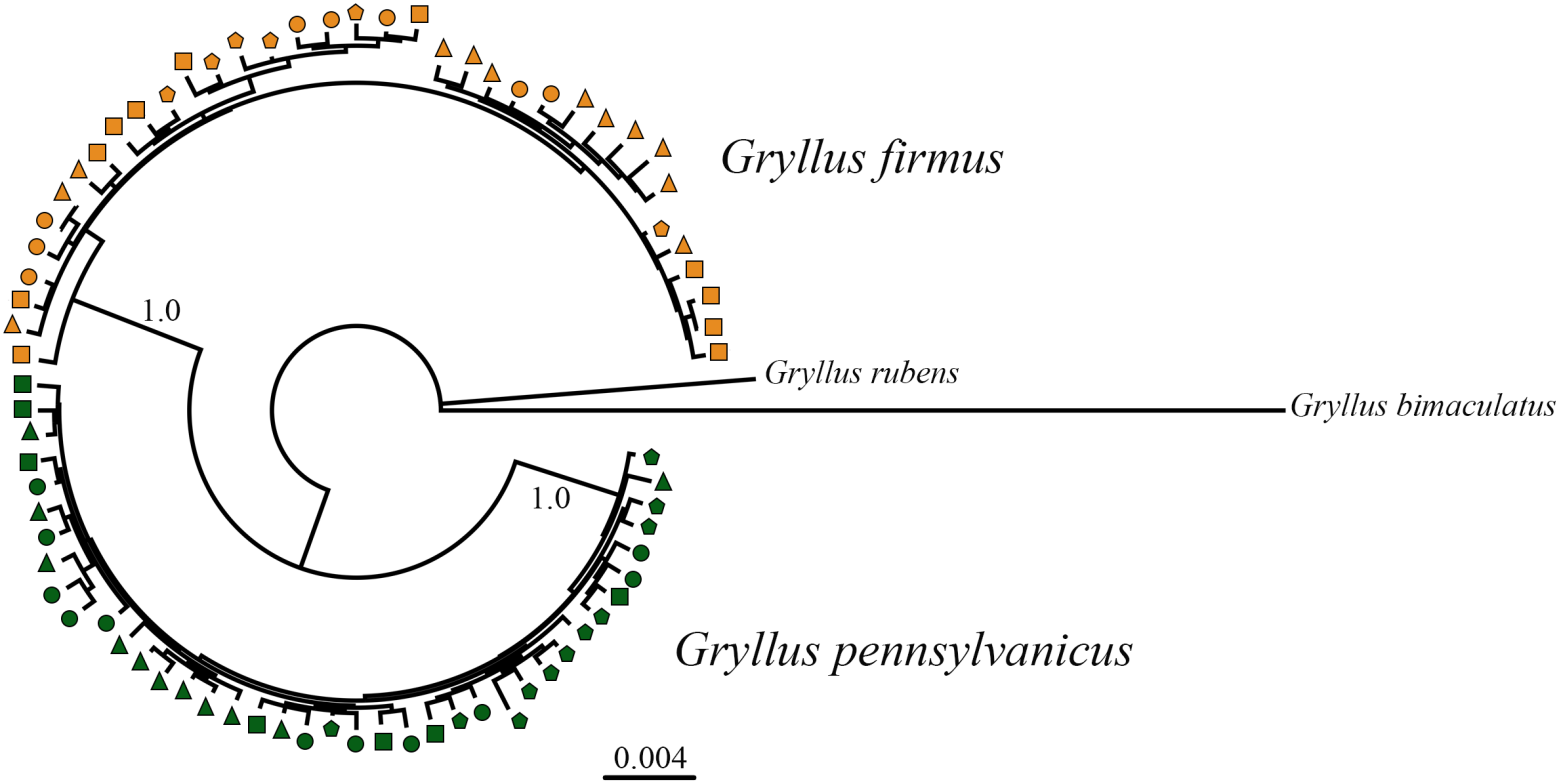
Bayesian phylogenetic tree for concatenated loci (172,357 nucleotides), showing populations for the two species. Other tree building methods also showed the two species as reciprocally monophyletic. Symbols indicate populations: GUI (yellow square), PAR (yellow triangle), TRI (yellow circle), JP (yellow pentagon), ITH (green square), SCO (green triangle), SCR (green circle) and POW (green pentagon).

The estimated mean divergence time was 819,119 years (with 95% Lo 601,306 to 95% hi 1,052,651 years). This divergence time is substantially larger than the previously estimated divergence time, based on mtDNA, of about 200,000 years (Maroja, Andres, and Harrison 2009; Willett, Ford, and Harrison 1997). We note that this divergence time is of course dependent on mutation rate calculation, in this case calibrated for each nuclear locus based on the divergence between the ingroups ( *G. firmus* / *G. pennsylvanicus* ) and outgroups ( *G. bimaculatus* and/or *G. rubens* ) sequences for each nuclear loci. The divergence time between ingroup and outgroups was initially calibrated with mtDNA and estimated at 3.2 million years for *G. bimaculatus* and 2.7 million years for *G. rubens* . When calibration is done based on divergence between *G. firmus* and *G. pennsylvanicus* (calibrated based on their mtDNA divergence time, 200 K years also according to the standard mtDNA mutation rate from Brower (1994)), then the nuclear mutation rate per locus is higher. However, with these estimated nuclear mutation rates (i.e., those calibrated with mtDNA divergence between *G. pennsylvanicus* and *G. firmus* ) the divergence with *G. bimaculatus* / *G. rubens* becomes much more recent than what is their mtDNA divergence suggests. This indicates that either mtDNA divergence has not been constant through the evolutionary history or has not been congruent with nuclear divergence. The discrepancy in mutation rates can be easily explained if mtDNA introgressed between *G. firmus* and *G. pennsylvanicus* . This explanation is supported by the unresolved mtDNA phylogeny for *G. firmus* and *G. pennsylvanicus* (Willett, Ford, and Harrison 1997), which contrasts with the nuclear DNA phylogeny (see Figure 4). For nuclear DNA, *G. firmus* and *G. pennsylvanicus* were reciprocally monophyletic with very strong branch support of 1.0 (identical results were observed with ML or distance trees).

The IMa3 program also estimates migration rates. The migration rate from *G. firmus* to *G. pennsylvanicus* was 0.1497 migrants per generation (2 N*m*) whereas the migration rate from *G. pennsylvanicus* into *G. firmus* was 0.0941 migrants per generation.

## 4 Discussion

We did not find evidence of introgression in allopatric populations of the *G. firmus* and *G. pennsylvanicus* system. While hybrids are certainly formed in the hybrid zone, and clines across the zone in both phenotypic (Ross and Harrison 2002) and genomic markers (Larson et al. 2014) exist, the genome of allopatric populations geographically close to the hybrid zone does not seem to conform to the “islands” model expectations (Cruickshank and Hahn 2014).

If the “islands model” holds, parts of the genome will be homogenized by gene flow between species, whereas other highly differentiated parts will be impermeable to gene flow due to divergent selection or because they contribute to reproductive barriers. Under this scenario we should expect to observe both high relative (*F*_ST_) and high absolute divergence (*D*_XY_) in the “impermeable” (non‐introgressing) regions. We instead found that genomic regions, previously characterized as “non‐introgressing,” in fact had lower (although not significantly so) absolute divergence than “introgressing” regions (Figure 3). This could be because introgression does not continue past the hybrid zone, and thus does not affect the genomes from allopatric population. However, it could suggest that, what previously had been assumed to be “islands of speciation,” might be “incidental islands” caused by selection and linked selection (Turner and Hahn 2010). It is possible that the genomic islands identified in the hybrid zone were a consequence of methodological caveats and hybridization patterns, and did not in fact represent loci under negative selection in hybrid offspring. When the introgression patterns were calculated (Larson, Andres, et al. 2013; Larson et al. 2014), the mapping location of SNPs was not yet known; therefore, X‐linked alleles in males were coded as “homozygotes” for the INTROGRESS (Gompert and Buerkle 2010) calculations—a methodological issue that could generate misleading patterns (also see Maroja et al. 2015). Furthermore, the one‐directional barrier to gene exchange, where male F1 offspring inherit the incompatibility towards *G. firmus* females, make introgression stronger from *G. firmus* into *G. pennsylvanicus* with reduced presence of *G. firmus* X chromosomes in relation to autosomes (since hybridizing *G. firmus* must be males (X0), F1 offspring will have more *G. pennsylvanicus* X chromosomes in a 2:1 ratio)—this would lead to a lack of introgression on the X compared to autosomes, leading to a presence of “islands” in the X (where basically the whole X behaves as an “island”).

### 4.1 Patterns of Differentiation Do Not Support a Simple “Islands of Differentiation” Model

To test the “islands” model, we sequenced a total of 48 introgressing and non‐introgressing loci originally developed from a male accessory gland transcriptome library (Andres et al. 2013; Larson, Andres, et al. 2013) that showed high allele frequency differences between two allopatric populations of each species (Guilford, CT and Ithaca, NY, Andres et al. 2013). Once mapped to the genome, these previously characterized markers were found to be represented in all cricket chromosomes (the 14 autosomes and the large X, representing ~20% of the genome, Lim, Vickery, and Mcekevan 1973—not all of these were sequenced here). However, the loci with steep clines classified as “non‐introgressing” (Larson, Andres, et al. 2013) and assumed to be impermeable to interspecific gene flow, were, for the most part, restricted to the X chromosome and a small region of autosome LG14 (Gainey, Kim, and Maroja 2018; Maroja et al. 2015), suggesting that genomic architecture might be important (Renaut et al. 2013). As a result of this clustered distribution of loci, our current sample of 48 loci was inevitably clumped; most of our “non‐introgressing” loci were X linked (*n* = 27, Figure 2) and the X contained very few “introgressing” loci (*n* = 4). Furthermore, two‐thirds (8/12) of the “non‐introgressing” autosomal loci were located to a small region of LG14. Therefore, disentangling the effects of X linkage from permeability across the hybrid zone was difficult.

Despite linkage difficulties, our results do not support an “islands” model. First, although *F*_ST_ was indeed higher for “non‐introgressing” loci, only four non‐introgressing loci are located on the X chromosome, and two of these loci (loci 8833 and 541, Figure 2 and DRYAD Table S3) were not able to be positioned in the second mapping study and therefore might not represent truly X linked loci (Gainey, Kim, and Maroja 2018). Second, when we restricted the analyses to autosomes, we found no *F*_ST_ differences between introgressing and non‐introgressing loci. Third, the *D*_XY_ results showed that “non‐introgressing” loci, were in fact less (although not significantly so) differentiated than “introgressing” loci, a result that directly contradicts expectations for the “islands” model, where we would expect that non‐introgressing loci would have higher relative and absolute genetic differentiation.

While we did not find any support for the island model, a few caveats should be considered. The characterization of loci as either “introgressing” or “non‐introgressing” might be an artifact (and since our population collection is allopatric, the current data cannot be used to recalculate clines). Our classification was based on two previous genomic cline studies conducted before the SNPs had been mapped (Larson, Andres, et al. 2013; Larson et al. 2014); therefore, the X‐linked loci could have been characterized erroneously as “non‐introgressing” because only female backcross could be interspecific heterozygotes (note that all males would have been coded as homozygotes, since it was unknown that they were in fact hemizygotes). However, even if this was the case and loci were indeed misclassified as introgressing or non‐introgressing, there was no correlation between *F*_ST_ and *D*_XY_, as we would expect if low *F*_ST_ (and *D*_XY_) were brought about by mixing of genomes through introgression.

Given that prezygotic isolation between these two species is strong (Harrison 1983, 1985; Larson, Becker, et al. 2013; Maroja et al. 2009, 2014), that gene flow is restricted on a fine scale (Larson et al. 2014; Ross and Harrison 2002), and that linkage disequilibrium is high even in the center of the hybrid zone (Harrison and Bogdanowicz 1997), it is also possible that introgression is indeed negligible and that the island model does not apply.

Our IMa3 calculations suggest a scenario of low migration (0.14–0.22 2 *N*_e_ *m*) and a divergence time of ~1.6 *N*_e_ generations. Assuming low migration, differences between “islands” and “genomic background” are apparent only after ≥ 0.4 N *N*_e_ generations (Cruickshank and Hahn 2014). Therefore, we should have been able to detect differences between these two models. However, if secondary contact is much more recent than the estimated divergence, it is possible that there has been no time for introgression to homogenize even the freely introgressing parts of the genome, and we may have no power to properly test the island's model.

### 4.2 The Importance of the X Chromosome

While our results put in question the “islands of divergence” model for the *G. firmus* and *G. pennsylvanicus* hybrid zone, they do not detract from the importance of the X chromosome in speciation (e.g., Charlesworth, Coyne, and Barton 1987; Good, Dean, and Nachman 2008; Hu and Filatov 2016; Masly and Presgraves 2007; Meisel and Connallon 2013) or to evidence of more intense selection on X‐linked loci, often associated with reduced introgression in other species (e.g., Carneiro et al. 2014; Fontaine et al. 2015; Garrigan et al. 2012; Hu and Filatov 2016; Payseur, Krenz, and Nachman 2004; Saetre et al. 2003; Sankararaman et al. 2014).

As is the case in many other systems, it is possible that the X chromosome is under stronger local selection or drift, bringing alleles to fixation and reducing ancestral polymorphism, increasing *F*_ST_ without necessarily increasing *D*_XY_. Our data show that the *G. firmus* X chromosome is indeed reduced in polymorphisms (*π*, Figure 3C), as would be expected due to its smaller effective population size (3/4N_e_) and/or more intense selection (recessive alleles exposed in males) and lower recombination in relation to autosomes. Furthermore, while *F*_ST_ is indeed higher for the X (although not significantly so, Figure 3A), suggesting more fixed variants due to selection or drift, the absolute genetic divergence (*D*_XY_) in the X is significantly lower than that in autosomes (Figure 3B). This contrasts to patterns observed in *Drosophila*, where the X seems to be *more* divergent than autosomes (Garrigan et al. 2015), but is concurrent with the pattern observed in human‐chimp comparisons (Dutheil et al. 2015; Narang and Wilson Sayres 2016). In lower divergence and polymorphism, the human‐chimp X chromosome is virtually devoid of incomplete lineage sorting (Dutheil et al. 2015). This pattern could be explained by more frequent recurrent selective sweeps (including the possibility of meiotic‐drive), in addition to higher background selection, leading to a depletion of polymorphism right before speciation (Dutheil et al. 2015). This hypothesis is further supported by the fact that divergence and polymorphism are lower around coding genes, but are closer to expected levels of variation (3/4 of autosome) when further away (Arbiza et al. 2014; Narang and Wilson Sayres 2016), as would be expected if the reduction of polymorphism was due to linked selection. If a similar reduction in variation happens in field crickets, then we would expect that the X would be less divergent due to the lack of variation at the onset of speciation.

The X chromosome has many unusual patterns and processes (Vicoso and Charlesworth 2006) and our data do not allow us to test particular models that can best explain the slightly higher X *F*_ST_—such as faster‐X evolution (Meisel and Connallon 2013), or dominance (Turelli and Orr 2000). However, as we have no evidence of hybrid male sterility in this system—F1 males cannot fertilize pure *G. firmus* females (one‐way incompatibility is maintained) but they are fertile with F1 females as well as pure *G. pennsylvanicus* —some of the models that assume hybrid male sterility should not apply to this system (Presgraves and Meiklejohn 2021).

It is also important to notice that the dynamics of the hybridization incompatibility have contributed to the X being characterized as “non‐introgressing” in the cricket hybrid zone. Because of the one‐way incompatibility (F1 hybrids can only be produced from *G. pennsylvanicus* females; Harrison 1983), the F1 generation will exhibit a 2:1 excess of *G. pennsylvanicus* X chromosomes (F1females: X_*penn*_X_*firm*_, F1 males: X_*penn*_0) and, *G. pennsylvanicus* males or male F1s (X_*penn*_0) cannot contribute to the introgression of X‐linked *G. firmus* alleles because they cannot produce backcross hybrids with *G. firmus* females (L. S. Maroja and E. L. Larson, unpublished data). In contrast, *G. firmus* autosomes are free to introgress into the *G. pennsylvanicus* background through the F1 males since they are fully fertile with *G. pennsylvanicus* females (Larson et al. 2012). Furthermore, if females behave in the wild as they do in lab, we would expect that F1 females would mate more promptly to *G. firmus* males, contributing to higher flow from *G. pennsylvanicus* into *G. firmus* (Maroja et al. 2009). Therefore, the mating dynamics of the hybrid zone, combined with the initial lack of linkage data for markers (males coded as homozygotes, see above) might help explain steeper X‐linked genomic clines observed across the two hybrid zone locations (Larson, Andres, et al. 2013; Larson et al. 2014)—this reduces the probability that the previously identified loci represent “barrier genes:” it is more likely they represent a genomic architecture phenomenon.

### 4.3 The Divergence Time Between Species

Based on mtDNA, *Gryllus firmus* and *G. pennsylvanicus* have been considered recently diverged species—the divergence time estimated to be only 200 K years (Broughton and Harrison 2003; Maroja, Andres, and Harrison 2009). For the first time, in this paper, we calculate the divergence time using nuclear DNA. Our estimate of ~800 K years makes the species divergence 4× older. This new estimate is consistent with the multiple described barriers to gene exchange between these two species (Maroja et al. 2009). This includes not only fertilization barriers (Harrison 1983; Larson et al. 2012), but cuticular hydrocarbon differences (Heggeseth et al. 2020; Maroja et al. 2014), time to mate (Harrison 1983), and habitat and temporal isolation (Ross and Harrison 2002). It is therefore likely that their divergence time has been longer than just 200 K years ago, especially given that throughout most of the species' range, they only have one generation per year.

A potential caveat of this new nuclear divergence time calculation is that it still relies on the mtDNA evolution rate (Brower 1994) for the estimation of initial mutation rate value for IMa3. Here, we used outgroups *G. bimaculatus* and *G. rubens* and obtained a mtDNA‐based estimated divergence time between *G. firmus* and *G. pennsylvanicus* and each outgroup. Then using this estimated time, we calculated a nuclear mutation rate between the outgroup and *G. firmus* / *G. pennsylvanicus* (DRYAD Table S1). Therefore, if the mutation rate estimate is incorrect, or if there are phylogenetic incongruencies between mtDNA and nuclear‐based trees (for the *G. firmus* /*G. pennsylvanicus*, *G. rubens*, and *G. bimaculatus* ), then our estimated mutation rates, and therefore calculated divergence time, would be compromised.

## 5 Conclusions

Our data do not support the “islands model”—that is, we have no evidence that parts of the genome are homogenized by gene flow, while other parts remain impermeable and highly differentiated due to divergent selection or because they act as barriers to gene exchange. These findings do not bring into question the importance of the X chromosome in hybridization and speciation—even allopatric species without hybrid zones often show selection against introgressed X. What we do demonstrate is that gene flow across the hybrid zone has not been sufficient to homogenize the genome of allopatric populations proximal to the hybrid zone; instead, most genes might follow a “non‐introgressing” model, with previously characterized non‐introgressing regions of the X chromosome exhibiting increased relative differentiation (*F*_ST_) possibly due to higher local selection and/or drift.

This result might not be as surprising in view of the longer divergence time. While according to mtDNA, the species divergence only 200 K years ago, our nuclear DNA estimates elevate this by 4×—it is therefore not as surprising that the crickets already have in place a number of barriers to gene exchange and that allopatric populations do not appear to show evidence of genomic mixing.

## Author Contributions

**Luana S. Maroja:** conceptualization (lead), data curation (equal), formal analysis (equal), funding acquisition (equal), investigation (equal), methodology (equal), project administration (lead), writing – original draft (equal), writing – review and editing (equal). **Francesca Barradale:** data curation (equal), formal analysis (equal), investigation (equal), methodology (equal), writing – original draft (equal). **Sebastian A. Espinoza‐Ulloa:** data curation (equal), formal analysis (equal). **Steven Bogdanowicz:** data curation (equal), formal analysis (equal), investigation (equal), methodology (equal). **Jose Andres:** conceptualization (equal), data curation (equal), formal analysis (equal), investigation (equal), methodology (equal), writing – original draft (equal), writing – review and editing (equal).

## Conflicts of Interest

The authors declare no conflicts of interest.

## Data Availability

Genetic data: Individual genotype data from the individually sequenced loci are available on DataDryad (https://doi.org/10.5061/dryad.s4mw6m9bb), this includes all FASTA files, IMa3 dataset as well as all data (*F*_ST_, *D*_XY_, pi, etc.) used in the R analyses (scripts also included). Additional figures and tables are also included in SuppDryad: (https://doi.org/10.5061/dryad.s4mw6m9bb). The annotated draft genome files (gff) from single *G. firmus* individual, are also available on DataDryad (https://doi.org/10.5061/dryad.s4mw6m9bb; Maroja et al. 2024). Genomic DNA data have been deposited in Genbank, accession numbers OR441320–OR447380 (Maroja et al. 2023).
